# The Mayo Endoscopic Score Is a Novel Predictive Indicator for Malignant Transformation in Ulcerative Colitis: A Long-Term Follow-Up Multicenter Study

**DOI:** 10.3389/fsurg.2022.832219

**Published:** 2022-03-16

**Authors:** Weimin Xu, Fangyuan Liu, Wenbo Tang, Yubei Gu, Jie Zhong, Long Cui, Peng Du

**Affiliations:** ^1^Department of Colorectal Surgery, Xinhua Hospital, Shanghai Jiaotong University School of Medicine, Shanghai, China; ^2^Department of Gastroenterology, Rui Jin Hospital, Affiliate to Shanghai Jiao Tong University, School of Medicine, Shanghai, China

**Keywords:** Mayo endoscopic score, ulcerative colitis, colorectal cancer, malignant transformation, colitis

## Abstract

**Background:**

Data on the relative risk of malignant transformation in ulcerative colitis (UC) are insufficient. We investigated the potential value of the Mayo endoscopic score (MES) for predicting malignant transformation in patients with UC.

**Methods:**

Data of patients with UC evaluated at our institute from June 1986 to December 2019 were retrospectively analyzed. The MES used in the study indicated the results of the first colonoscopy after hospitalization. We defined MES of 0–1 as low and MES of 2–3 as high. Univariable and multivariate logistic regression models were used for statistical analysis.

**Results:**

Among the 280 eligible patients with UC with a median follow-up time of 14 (interquartile range, 10.0–18.0) years, those with a high MES were more likely to develop malignant transformation. High MES positively correlated with the degree of malignancy and was an independent risk factor for UC-associated dysplasia and colorectal cancer (CRC, odds ratio [OR], 9.223; 95% confidence interval [CI], 1.160–73.323; *p* = 0.036). Disease duration >5 years (OR, 2.05; 95% CI, 1.177–3.572; *p* = 0.011), immunomodulator use (OR, 4.314; 95% CI, 1.725–10.785; *p* = 0.002), biologics nonuse (OR, 3.901; 95%CI, 2.213–6.876; *p* < 0.001), and Hb <90 g/L (OR, 2.691; 95% CI, 1.251–5.785; *p* = 0.011) were contributing factors for high MES.

**Conclusion:**

High MES could be a novel predictor of malignant transformation in UC. Clinicians should optimize the use of biologics and immunomodulators early and should actively correct anemia to improve the MES and then reduce the incidence of UC-associated dysplasia and CRC.

## Introduction

Ulcerative colitis (UC) is a major form of inflammatory bowel disease (IBD) characterized by chronic recurrent inflammation. Previous studies have demonstrated that the disease duration of UC is a definite risk factor for colorectal neoplasia (1), which was the most serious complication in UC. Approximately 25–30% of patients with UC ultimately require colectomy due to UC-associated neoplasia (2).

Although intestinal inflammation was associated with UC-associated neoplasia, few studies have reported whether colonoscopy assessment could predict the occurrence of malignant transformation. Therefore, early assessment of the possibility of malignant transformation in UC through endoscopic evaluation is becoming increasingly important. There are several indices for the measurement of endoscopic severity in UC (3), including the Mayo endoscopic score (MES) and the Ulcerative Colitis Endoscopic Index of Severity (UCEIS).

Although the latter is more accurate in assessing disease activity and predicting prognosis (4, 5), the MES is still more popular with clinicians and is widely used to evaluate the disease activity in UC because of its convenience and applicability in clinical decision-making (4, 6). However, few studies have reported the relationship between the MES and malignant transformation in UC.

This study aimed to assess the clinical outcomes of patients with different MES according to the first colonoscopy after hospitalization to demonstrate whether the MES endoscopic evaluation system is associated with malignant transformation in UC. Additionally, we explored the risk factors for high MES, which could facilitate appropriate actions by clinicians to prevent high MES to reduce the possibility of UC-associated dysplasia and, thereby, the incidence of colorectal cancer (CRC). Hence, accurate and early colonoscopy after hospitalization should be recommended for the definitive evaluation of the MES.

## Materials and Methods

### Study Population

We retrospectively collected and reviewed the clinical data of patients with UC who were evaluated from June 1986 to December 2019 at two hospitals (Xinhua and Ruijin hospitals) in Shanghai, China. The clinical data of the patients were obtained from a prospectively maintained Institutional Review Board-approved database (Chinese Database System for IBD) as we previously mentioned (7, 8). Patients with UC aged more than 18 years with regular follow-up were included in this study, whereas patients diagnosed with Crohn's disease (CD) or familial adenomatous polyposis (FAP) and those with poor compliance were excluded.

### MES Evaluation

The MES used in the study indicated the result of the first colonoscopy after hospitalization. In this study, the MES score was assessed through electronic photographs of the medical record system, and earlier endoscopic data were through the printed colonoscopy report. Two independent endoscopists who were blinded to our research were invited to evaluate the MES scores. When there was inconsistency in the MES scores of a patient recorded by the two evaluators, the higher value was chosen for our analysis. According to previous research, the MES was classified into four (0–3) categories based on the endoscopic findings, such as erosions, vascular pattern, erythema, friability, and ulceration. In this study, we defined scores of 0–1 as low MES and scores of 2–3 as high MES.

### Clinical Data Evaluation

Clinical data of all patients were collected from the Chinese Database System for IBD. We contacted the patients by phone and asked them to come to the hospital for colonoscopy and outpatient follow-up every year. If there was any disease, biopsy would be taken in time for pathological examination. The diagnosis of UC was strictly based on endoscopic and histologic examinations. The extent of UC was classified according to the Montreal classification system (9). We evaluated the severity of colitis histopathologically according to the methods reported in previous research (10). The histopathological scores of colitis were assessed based on neutrophils infiltration, crypts, crosssection involvement, and erosion or ulceration formation by hematoxylin–eosin staining on a scale of 0–3. Histopathology evaluation and review were conducted independently by two independent pathologists. In this study, colitis-associated dysplasia refers to a malignant transformation that occurs under long-term intestinal inflammatory conditions. Intestinal obstruction, colon perforation, or serious gastrointestinal bleeding during hospitalization and follow-up were defined as serious complications. UC-associated dysplasia or CRC diagnosis was ultimately based on pathological results. Mesalamine, biologics, steroids, and immunomodulators refereed to those used in other hospitals before undergoing treatment in our institution. Previous surgery unrelated to UC was considered in the history of surgery. Weight loss in this study was defined as losing more than 5 kg during the disease duration. The colorectal stricture was diagnosed according to the results of endoscopic and surgical specimens (7).

### Statistical Analysis

We used unpaired Student's *t*-test, the chi-squared or Fisher's exact test, and Wilcoxon's rank-sum test to compare different variables. Multivariate logistic regression analyses were performed to identify the significant variables and to further determine the risk factors for malignant transformation and high MES. Statistical Package of the Social Sciences 19.0 (SPSS, IBM 2010, Chicago, IL, USA) and GraphPad Prism 5 Software (San Diego, CA, USA) were used for all statistical analyses. In this study, all hypothesis tests were two-sided with confidence intervals (CI) set at 95%, and a *p*-value of < 0.05 was considered statistically significant.

### Ethical Considerations

Patients provided signed informed consent for the patient's case data and surgical specimens obtained for this study. The Ethics Committee has reviewed the study principle and process as well as the application form. The Ethics Committee of our hospital approved this study (Approval No. XHEC-D-2020-020).

### English Editing

The English language of the paper was edited by a certified professional editor.

## Results

### Patients' Characteristics

**Figure 1:**
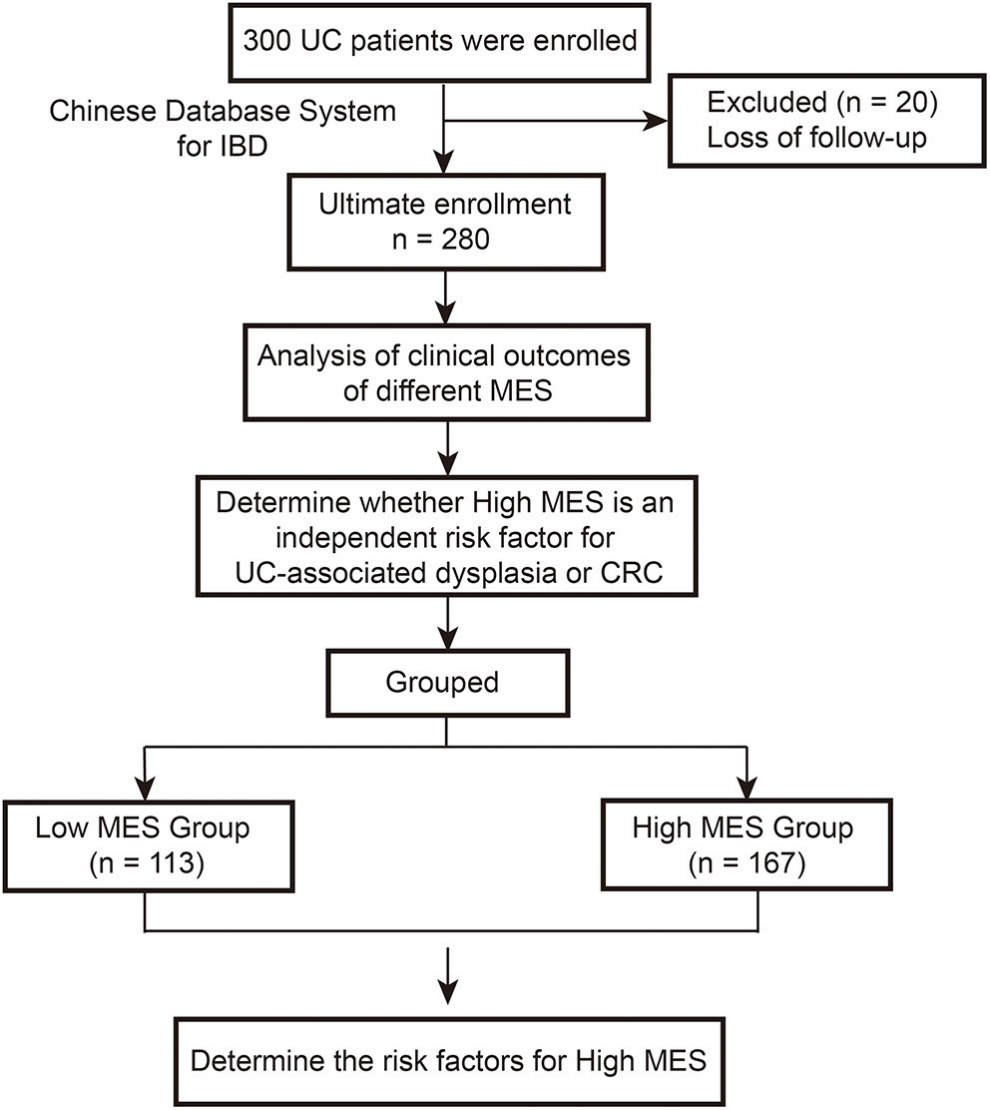
Flow chart of the study methodology.

As shown in the flow chart of this study (Figure 1), 300 patients with UC were evaluated at our institute from June 1986 to December 2019. Ultimately, a total of 280 eligible patients were included in the current study after excluding 20 patients lost to follow-up.

**Figure 2:**
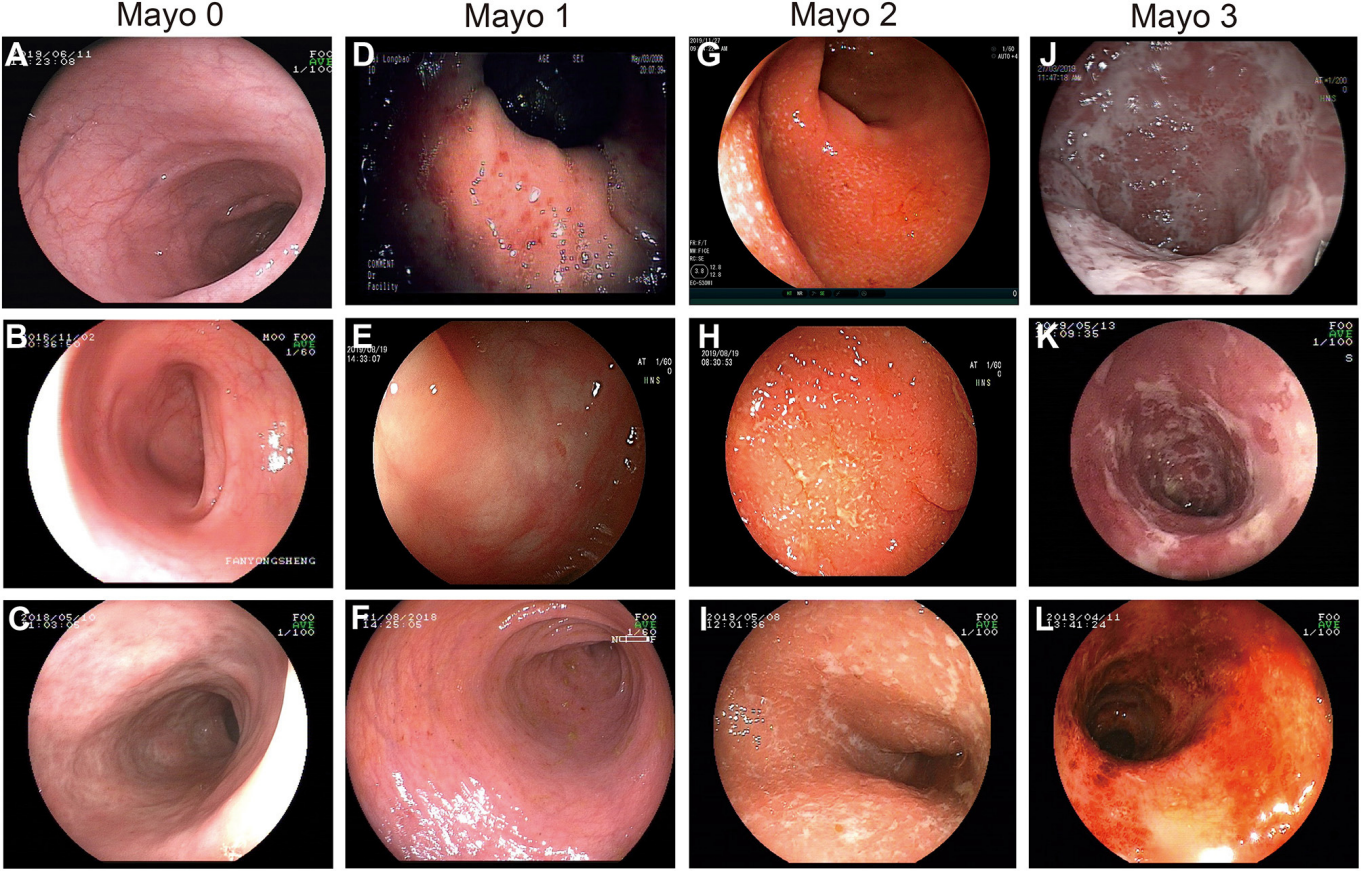
Endoscopic images demonstrating the MES scoring system. **(A–C)** MES of 0: normal or inactive disease. **(D–F)** MES of 1: **(D)** mild disease with erythema, **(E)** decreased vascular patterns, **(F)** mild erosion. **(G–I)** MES of 2: absence of vascular patterns, and erosions. **(J–L)** MES of 3: **(J,K)** serious ulceration and **(L)** spontaneous bleeding.

The whole cohort had a median follow-up time of 14 [interquartile range (IQR): 10.0–18.0) years and a median age at diagnosis of 42 (IQR: 29.0–53.0) years. Among all, 25 patients (8.9%) had an MES of 0, 88 (31.5%) had 1, 81 (28.9%) had 2, and 86 (30.7%) had 3. The detailed MES scoring system and endoscopic images for different MES according to the first colonoscopy after hospitalization are shown in Figure 2. Additionally, 7 patients (2.5%) were diagnosed with proctitis (E1), 144 (51.4%) developed left-sided colitis (E2), and 129 (46.1%) underwent pancolitis (E3). The detailed clinical data are shown in Table 1.

**Table 1:** Main baseline characteristics of patients.

**Variables**	**All cases (*n* = 280)**
Sex (male/female)	137/143
Age at diagnosis [yr, median (IQR)]	42.0 (29.0–53.0)
Disease duration [yr, median (IQR)]	6.5 (4.0–10.0)
Follow-up time [yr, median (IQR)]	14.0 (10.0–18.0)
MES, *n* (%)	
0	25 (8.9)
1	88 (31.5)
2	81 (28.9)
3	86 (30.7)
**Conditions of relapse**, ***n*** **(%)**	
First occurrence	63 (22.50)
First recurrence	48 (17.1)
Multiple recurrence	169 (60.4)
**Stool frequency**, ***n*** **(%)**	
<4 times	225 (80.4)
≥4 time	55 (19.6)
Weight loss, *n* (%)	106 (37.9)
Extraintestinal manifestation (EIM), *n* (%)	
No	248 (88.6)
Yes	32 (11.4)
History of surgery, *n* (%)	40 (14.3)
Family history, *n* (%)	18 (6.4)
Family history of CRC	4 (1.4)
Family history of IBD	3 (1.1)
Family history of other caner	11 (3.9)
**Extent of UC**, ***n*** **(%)**	
E1	7 (2.5)
E2	144 (51.4)
E3	129 (46.1)
Mesalamine, *n* (%)	167 (59.6)
Biologics, *n* (%)	97 (34.6)
Steroids, *n* (%)	173 (61.8)
Immunomodulators, *n* (%)	39 (13.9)
Hb (g/L, mean ± SD)	113.9 ± 25.2
Alb (g/L, mean ± SD)	28.4 ± 21.8

*MES, Mayo endoscopic score; IQR, Interquartile range; CRC, Colorectal cancer; IBD, Inflammatory bowel disease; UC, Ulcerative colitis; Hb, hemoglobin; Alb, Albumin*.

### Complications Analysis

A total of 110 patients (39.3%) from the entire cohort developed complications during hospitalization and follow-up. Of those, 72 patients (25.7%) developed inflammatory polyps, which was the most common complication in this study. In addition, serious gastrointestinal bleeding, intestinal obstruction, and colon perforation occurred in 28 (10.0%), 21 (7.5%), and 6 patients (2.1%), respectively. Moreover, 21 patients (7.5%) developed malignant transformation. Among them, 10 (3.6%) and 11 (3.9%) patients were diagnosed with UC-associated dysplasia and CRC, respectively (Supplementary Table 1). In this study, all dysplasia refers to high-grade dysplasia according to pathological results. Patients with high-grade dysplasia were recommended to receive colectomy to prevent the development of CRC.

### Comparison of the Clinical Outcomes Between the low MES and High MES Groups

**Figure 3:**
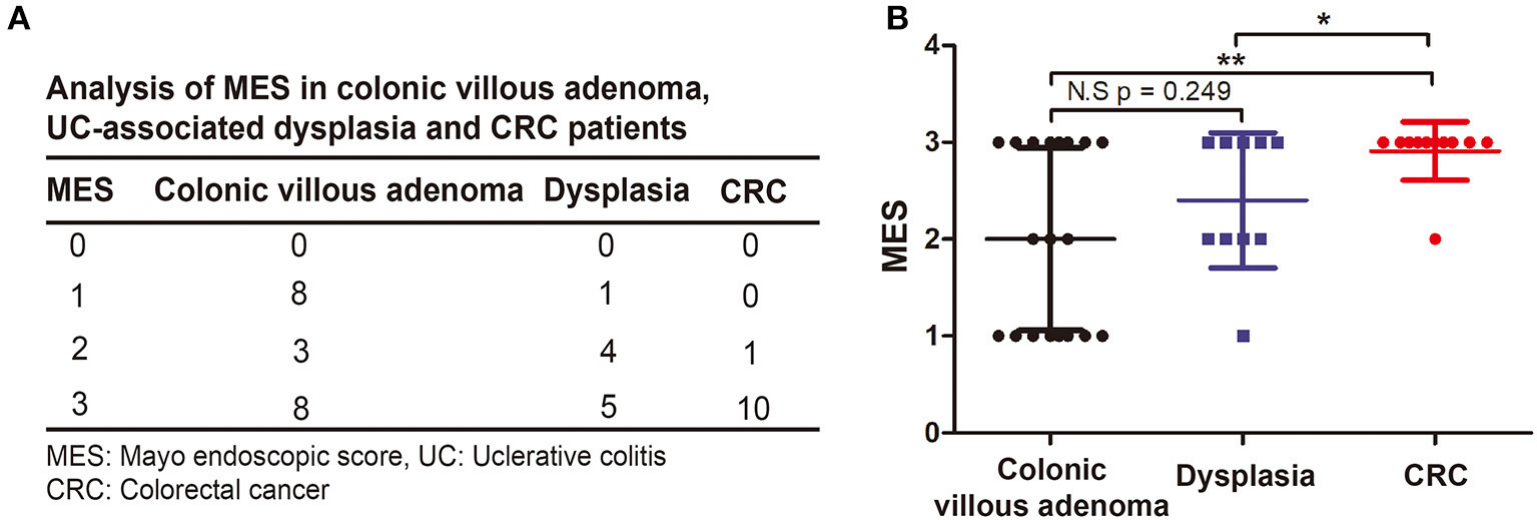
Comparison the association between MES and degree of malignancy. **(A)** Descriptive analysis of the distribution of MES in colonic villous adenoma, UC-associated dysplasia and CRC. **(B)** Two-sample Student's t-test was performed to analyze the association between MES and degree of malignancy.

To determine whether the MES can predict the formation of UC-associated dysplasia and CRC, we compared the clinical outcomes between patients with low and high MES. As shown in Table 2, we first found that remission (*p* = 0.024) was more common in patients with low MES than in those with high MES. Patients with high MES were prone to have active disease (*p* = 0.033), develop serious complications (*p* < 0.001), and undergo surgery (*p* < 0.001) than those in the low MES group. Additionally, we found that 20 patients (95.2%) with malignant transformation had a MES of 2 or 3 (*p* = 0.001). We then compared the MES in 19 cases of colonic villous adenoma, 10 cases of dysplasia, and 11 cases of CRC (Figure 3A) and found that the MES progressively increased from precancerous lesions to dysplasia and intestinal cancer (Figure 3B). Collectively, these data indicated that a high MES was associated with malignant transformation. Moreover, a higher MES was associated with an increasing degree of malignancy. Therefore, we speculated that a high MES could be a novel predictor of malignant transformation in UC.

**Table 2:** Comparison of clinical outcomes between patients with UC with low MES and high MES group.

**Variables**	**Low MES group**	**High MES group**	***p* value**
Remission, *n* (%)			0.024^a^
No	46 (33.6)	91 (66.4)	
Yes	67 (46.9)	76 (53.1)	
Alternate or continuous disease activity, *n* (%)			0.033^a^
No	89 (44.3)	112 (55.7)	
Yes	24 (30.4)	55 (69.6)	
Serious complications, *n* (%)			<0.001^a^
No	110 (46.2)	128 (53.8)	
Yes	3 (7.1)	39 (92.9)	
Surgery, *n* (%)			<0.001^a^
No	98 (58.0)	71 (42.0)	
Yes	15 (13.5)	96 (86.5)	
Malignant transformation, *n* (%)			0.001^a^
No	112 (99.1)	1 (0.9)	
Yes	1 (4.8)	20 (95.2)	
Death, *n* (%)			0.053^b^
No	112 (41.5)	158 (58.5)	
Yes	1 (10.0)	9 (90.0)	

a *Chi-squared*.

b *Fisher's exact test*.

*Serious complications, Intestinal obstruction, colon perforation and gastrointestinal bleeding. MES, Mayo endoscopic score; UC, Ulcerative colitis*.

### High-MES Was an Independent Risk Factor for Malignant Transformation in UC

To further investigate whether a high MES was an independent risk factor for malignant transformation, we found that age at diagnosis (*p* = 0.017), disease duration (*p* = 0.014), MES (*p* = 0.001), and colorectal stricture (*p* < 0.001) were significantly associated with malignant transformation in the univariable analysis (Table 3). As shown in Table 3, according to the results of a multivariate logistic regression analysis, age more than 40 years at diagnosis [odds ratio (OR), 4.436; 95% CI, 1.305–15.071, *p* = 0.017], high MES (OR, 9.223; 95% CI, 1.160–73.323; *p* = 0.036), and colorectal stricture (OR, 7.346; 95% CI, 2.595–20.796; *p* < 0.001) were contributing factors for malignant transformation in UC.

**Table 3:** Univariable analysis and multivariate logistic regression analysis of risk factors for malignant transformation in UC.

**Variables**	**Non-malignant group**	**Malignant Group**	**Univariate analysis**	**Multivariate logistic regression analysis**		
			***p*-value**	**Odds ratio**	**95% CI**	***p*-value**
Sex, *n* (%)			0.901^a^			
Male	127 (92.7)	10 (7.3)				
Female	132 (92.3)	11 (7.7)				
Age at diagnosis, *n* (%)			0.017^a^			
<40y	119 (96.7)	4 (3.3)				
≥40y	149 (89.8)	17 (10.2)		4.436	1.305-15.071	0.017
Disease duration, *n* (%)			0.014^a^			
<10y	189 (95.0)	10 (5.0)				
≥10y	70 (86.4)	11 (13.6)		1.989	0.726-5.453	0.181
MES, *n* (%)			0.001^a^			
Low MES	112 (99.1)	1 (0.9)				
High MES	147 (88.0)	20 (12.0)		9.223	1.160-73.323	0.036
Conditions of relapse, *n* (%)			0.68^b^			
First occurrence	58 (92.1)	5 (7.9)				
First recurrence	46 (95.8)	2 (4.2)				
Multiple recurrence	155 (91.7)	14 (8.3)				
EIM, *n* (%)			0.278^c^			
No	231 (93.1)	17 (6.9)				
Yes	28 (87.5)	4 (12.5)				
Colorectal stricture, *n* (%)			<0.001^a^			
No	233 (95.9)	10 (4.1)				
Yes	26 (70.3)	11 (29.7)		7.346	2.595-20.796	<0.001
Weight loss, *n* (%)			0.981^a^			
No	161 (92.5)	13 (7.5)				
Yes	98 (92.5)	8 (7.5)				
History of surgery, *n* (%)			0.095^c^			
No	225 (93.8)	15 (6.2)				
Yes	34 (85.0)	6 (15.0)				
Family history, *n* (%)			<0.001^a^			
No	246 (93.9)	16 (6.1)				
CRC or IBD	2 (28.6)	5 (71.4)		1.388	2.595–20.796	0.466
Other caner	11 (100.0)	0 (0.0)				
Extent of UC, *n* (%)			0.643^b^			
E1	6 (85.7)	1 (14.3)				
E2	135 (93.8)	9 (6.2)				
E3	118 (91.5)	11 (8.5)				
Hb, *n* (%)			0.77^c^			
≥90 g /L	214 (92.6)	17 (7.4)				
<90 g/L	45 (91.8)	4 (8.2)				
Alb, *n* (%)			0.59^a^			
≥35 g/L	89 (93.7)	6 (6.3)				
<35 g/L	170 (91.9)	15 (8.1)				

a *Chi-squared*.

b *Wilcoxon's rank-sum test*.

c *Fisher's exact test. CI, Confidence interval. UC, Ulcerative colitis; MES, Mayo endoscopic score; EIM, Extraintestinal manifestation; CRC, Colorectal cancer; IBD, Inflammatory bowel disease; Hb, hemoglobin; Alb, Albumin*.

### Risk Factors for High-MES

The above results indicated that patients with high pre-ileal pouch–anal anastomosis (IPAA) MES were more likely to develop UC-associated dysplasia and CRC. Therefore, we further explored the factors that can contribute to high pre-IPAA MES in UC. The results of the univariate analysis indicated that disease duration (*p* = 0.041), history of surgery (*p* = 0.032), use of immunomodulators (*p* = 0.002), use of biologics (*p* < 0.001), and the level of Hb (*p* = 0.012) were significantly associated with MES (Table 4). The multivariate logistic regression analysis indicated that disease duration of more than 5 years (OR, 2.05; 95% CI, 1.177–3.572; *p* = 0.011), previous use of immunomodulators (OR, 4.314; 95% CI, 1.725–10.785; *p* = 0.002), nonuse of biologics (OR, 3.901; 95% CI, 2.213–6.876; *p* < 0.001), and a Hb level of <90 g/L (OR, 2.691; 95% CI, 1.251–5.785; *p* = 0.011) were independent risk factors for high MES (Table 4). The results are also demonstrated in Supplementary Figure 1, which shows that patients with a disease duration of more than 5 years, those who previously used immunomodulators, had a Hb level of <90 g/L after hospitalization, or did not use biologics had a higher MES.

**Table 4:** Univariable analysis and multivariate logistic regression analysis of risk factors for high MES in UC.

**Variables**	**Low MES group**	**High Mes Group**	**Univariate analyses**	**Multivariate logistic regression analysis**		
			***p*-value**	**Odds Ratio**	**95% CI**	***p*-value**
Sex, *n* (%)			0.577^a^			
Male	53 (38.7%)	84 (61.3%)				
Female	60 (42.0%)	83 (58.0%)				
Age at diagnosis, *n* (%)			0.929^a^			
<40y	50 (40.7%)	73 (59.3%)				
≥40y	63 (40.1%)	94 (59.9%)				
Disease duration, *n* (%)			0.041^a^			
<5y	45 (48.9%)	47 (51.1%)				
≥5y	68 (36.2%)	120 (63.8%)		2.05	1.177-3.572	0.011
Conditions of relapse, *n* (%)			0.131^b^			
First occurrence	33 (52.4%)	30 (47.6%)				
First recurrence	16 (33.3%)	32 (66.7%)				
Multiple recurrence	64 (37.9%)	105 (62.1%)				
Weight loss, *n* (%)			0.089^a^			
No	77 (44.3%)	97 (55.7%)				
Yes	36 (34.0%)	70 (56.0%)	0.032^a^			
**History of surgery**, ***n*** **(%)**
No	103 (42.9%)	137 (57.1%)				
Yes	10 (25.0%)	30 (75.0%)		1.581	0.704-3.552	0.267
Family history, *n* (%)			0.531^a^			
No	108 (41.2%)	154 (58.8%)				
CRC or IBD	2 (28.6%)	5 (71.4%)				
Other caner	3 (27.3%)	8 (72.7%)				
Extent of UC, *n* (%)			0.002^b^			
E1	3 (42.9%)	4 (57.1%)				
E2	71 (49.3%)	73 (59.7%)				
E3	39 (30.2%)	90 (69.8%)				
Steroids, *n* (%)			0.050^a^			
No	51 (47.7%)	56 (52.3%)				
Yes	62 (35.8%)	111 (64.2%)				
Immunomodulators, *n* (%)			0.002^a^			
No	106 (44.0%)	135 (56.0%)				
Yes	7 (17.9%)	32 (82.1%)		4.314	1.725-10.785	0.002
Biologics, *n* (%)			<0.001^a^			
No	57 (31.1%)	126 (68.9%)		3.901	2.213-6.876	<0.001
Yes	56 (57.7%)	41 (42.3%)				
Hb, *n* (%)			0.012^a^			
≥90 g /L	101 (43.7%)	130 (56.3%)				
<90 g/L	12 (24.5%)	37 (75.5%)		2.691	1.251-5.785	0.011
Alb, *n* (%)			0.059^a^			
≥35 g/L	31 (32.6%)	64 (67.4%)				
<35 g/L	82 (44.3%)	103 (55.7%)				

a *Chi-squared*.

b *Wilcoxon's rank-sum test*.

*UC, Ulcerative colitis; MES, Mayo endoscopic score; EIM, Extraintestinal manifestation; CRC, Colorectal cancer; IBD, Inflammatory bowel disease; Hb, hemoglobin; Alb, Albumin*.

## Discussion

The current research is the first to indicate that an early-diagnosed high MES (MES of 2 and 3) can predict and contribute to malignant transformation in UC. We further demonstrated that disease duration of more than 5 years, previous use of immunomodulators, nonuse of biologics, and a Hb level of <90 g/L were independent risk factors for high pre-IPAA MES. These results will help clinicians to undertake interventions to prevent high MES, thereby reducing the incidence of malignant transformation. An accurate and early assessment of MES based on the first colonoscopy after hospitalization should be recommended for the clinicians.

Ulcerative colitis-associated dysplasia and CRC seriously compromise the prognosis of patients with UC. Although colonoscopic surveillance was recommended by the American and British guidelines (11, 12) to be routinely performed to detect potential dysplasia or CRC, a previous study indicated that ~20% of patients with dysplasia and CRC were missed according to the recommended guidelines (13). Therefore, it remains a challenge for clinicians to predict early malignant transformation in UC by colonoscopic surveillance. Exploring novel indicators that can predict the malignant transformation of UC *via* colonoscopy and early prediction of the possibility of malignant transformation of UC are important research issues in UC management.

The MES is widely used to describe the degree of endoscopic activity in UC colonoscopy. An MES of 0 or 1 is usually considered as mucosal healing. However, recent studies have reported that mucin depletion in patients with an MES of 0 and lack of therapeutic intervention in patients with an MES of 1 were both associated with disease relapse (14, 15). A high MES (MES of 2 and 3) is generally considered an indicator of disease activity in UC, while its relationship with malignant transformation has not been reported. In the present study, we compared the clinical outcomes between patients with a low MES and a high MES and found that 95.2% of patients who developed malignant transformation had a high MES and that the MES was positively correlated with the degree of malignant transformation. In consideration of the results of the multivariate logistic regression analysis for malignant transformation, we first proposed that a high MES was a novel approach to predict malignant transformation in UC.

In the multivariate logistic regression analysis, we found age at diagnosis of more than 40 years to be an independent risk factor for malignant transformation in UC. Patients with disease onset over 40 years in the present study mean partly with the duration of inflammatory disease. This result was also found in previous research which indicated that there is a positive correlation between the cumulative risk of neoplasia and the duration of the disease (1). A more accurate correlation between the duration of disease and the risk of cancer needs further research with larger samples.

Itzkowitz demonstrated that the sequence of inflammation–dysplasia–carcinoma was an important process of UC-associated dysplasia and CRC (16), which indicated that intestinal inflammation was a contributing factor for malignant transformation in UC. Patients with a high MES were in the active period of UC with severe intestinal inflammation and were more likely to develop chronic inflammation with recurrent relapse, contributing to the formation of inflammatory polyps and colorectal stricture. A previous study reported that inflammatory polyps and colorectal stricture were contributing factors for CRC in UC (7). Therefore, a high MES could be an effective predictor of UC-associated dysplasia and CRC. We further indicated that disease duration of more than 5 years, a Hb level of <90 g/L, use of immunomodulators, and nonuse of biologics can contribute to high MES.

Bergamaschi et al. reported that anemia was associated with high levels of erythrocyte sedimentation rate (ESR) and C-reactive protein (CRP) (17). A nationwide crosssectional study indicated that disease activity and the use of corticosteroids could contribute to the development of anemia (18). A retrospective study by Madanchi et al. indicated that CD patients with anemia had a higher Crohn's disease activity index, while patients with UC were more likely to develop disease activity-associated erythema nodosum (19). This result was consistent with a previous study which indicated that anemia was associated with developing extraintestinal manifestation (EIM) in UC (8). Therefore, the increased disease activity leads to anemia, which in turn aggravates the disease activity, creating an inflammatory cycle that causes an increase in the MES.

Motoya et al. recently demonstrated that patients with UC and anemia who received concomitant immunosuppressant medications were more likely to develop adverse events in monocyte adsorptive apheresis (20). In a previous study, it was also reported that reasonable selection of an immunomodulator therapy course could improve the long-term quality of life of patients with UC after pouch surgery (21). Therefore, massive and irregular use of immunomodulators could lead to adverse outcomes.

In recent years, targeted therapy represented by tumor necrosis factor (TNF) antagonists has achieved good efficacy in the treatment of IBD. Most recently, the TNF antagonist ustekinumab was demonstrated as an effective induction and maintenance therapy for moderate-to-severe UC (22). In addition, previous research indicated that anti-TNF-α therapy can significantly induce the resolution of anemia and control disease activity (23). Gaetano Bergamaschi et al. indicated that infliximab could increase the growth of erythroid progenitors from the peripheral blood of patients with active disease to improve anemia (17), while Shu et al. discovered that infliximab blocked the caspase-3/8 and NF-κB pathways and inhibited the expression of hepcidin, a key regulator of iron metabolism, to mediate the occurrence of anemia in IBD (24). In summary, we speculate that biologics may control disease activity by inhibiting the inflammatory response and improving anemia.

There are some limitations in the present study. First, it was a retrospective study; thus, patients lost to follow-up were inevitable. Second, the sample of patients with full clinical and follow-up information at our institution was relatively small. Further multicenter studies with larger samples are required to confirm the results.

## Conclusion

In the present study, our results indicated that patients with UC who had a high MES recorded by the first colonoscopy after hospitalization were more likely to develop malignant transformation. Furthermore, a higher MES was associated with an increasing degree of malignancy. A high MES was a significant contributing factor for UC-associated dysplasia and CRC. We first concluded that a high MES could be a novel and effective predictor of malignant transformation in UC.

Moreover, we demonstrated that disease duration of more than 5 years, previous use of immunomodulators, nonuse of biologics, and a Hb level of <90 g/L after hospitalization were independent risk factors for high MES. Therefore, appropriate and effective use of biologics to control anemia and a reasonable selection of immunomodulators should be considered to improve the MES and to reduce the possibility of malignant transformation, thereby improving the long-term prognosis of patients with UC. Hence, accurate and early colonoscopy after hospitalization should be recommended for definitive evaluation of the MES.

## Data Availability Statement

The original contributions presented in the study are included in the article/Supplementary Material, further inquiries can be directed to the corresponding authors.

## Author Contributions

PD, LC, and YG conceived the study. WX analyzed the data and wrote the manuscript. FL and WT assisted in some analysis. JZ collaborated to collect the information of patients. All authors participated in revising the manuscript and approved the final version.

## Funding

This work was supported by the National Key R&D Program of China (2019YFC1316002), the National Natural Science Foundation of China (No. 82000481), and the Shanghai Sailing Program (No. 20YF1429400).

## Conflict of Interest

The authors declare that the research was conducted in the absence of any commercial or financial relationships that could be construed as a potential conflict of interest.

## Publisher's Note

All claims expressed in this article are solely those of the authors and do not necessarily represent those of their affiliated organizations, or those of the publisher, the editors and the reviewers. Any product that may be evaluated in this article, or claim that may be made by its manufacturer, is not guaranteed or endorsed by the publisher.
