# Supplementary material for: The Mayo Endoscopic Score Is a Novel Predictive Indicator for Malignant Transformation in Ulcerative Colitis: A Long-Term Follow-Up Multicenter Study
Source: Front Surg. 2022 Mar 16;9:832219. doi: 10.3389/fsurg.2022.832219 (PMC8965860; doi:10.3389/fsurg.2022.832219)
Supplement: Supplementary file 1 [file Data_Sheet_1.docx]

| **Supplementary Table 1. Analysis of complications in UC patients** | |
| --- | --- |
| **Complications** | **N (%)** |
| Colorectal stricture | 37 (13.2) |
| Serious Bleeding | 28 (10.0) |
| Inflammatory polyps | 72 (25.7) |
| Intestinal obstruction | 21(7.5) |
| Colon perforation | 6 (2.1) |
| Toxic megacolon | 2 (0.7) |
| CD | 2 (0.7) |
| Abscess formation | 3 (1.4) |
| Malignant transformation | 21 (7.5) |
| UC-associated dysplasia | 10 (3.6) |
| UC-associated CRC | 11 (3.9) |

CD, Crohn’s disease; UC, Ulcerative colitis; CRC, Colorectal cancer


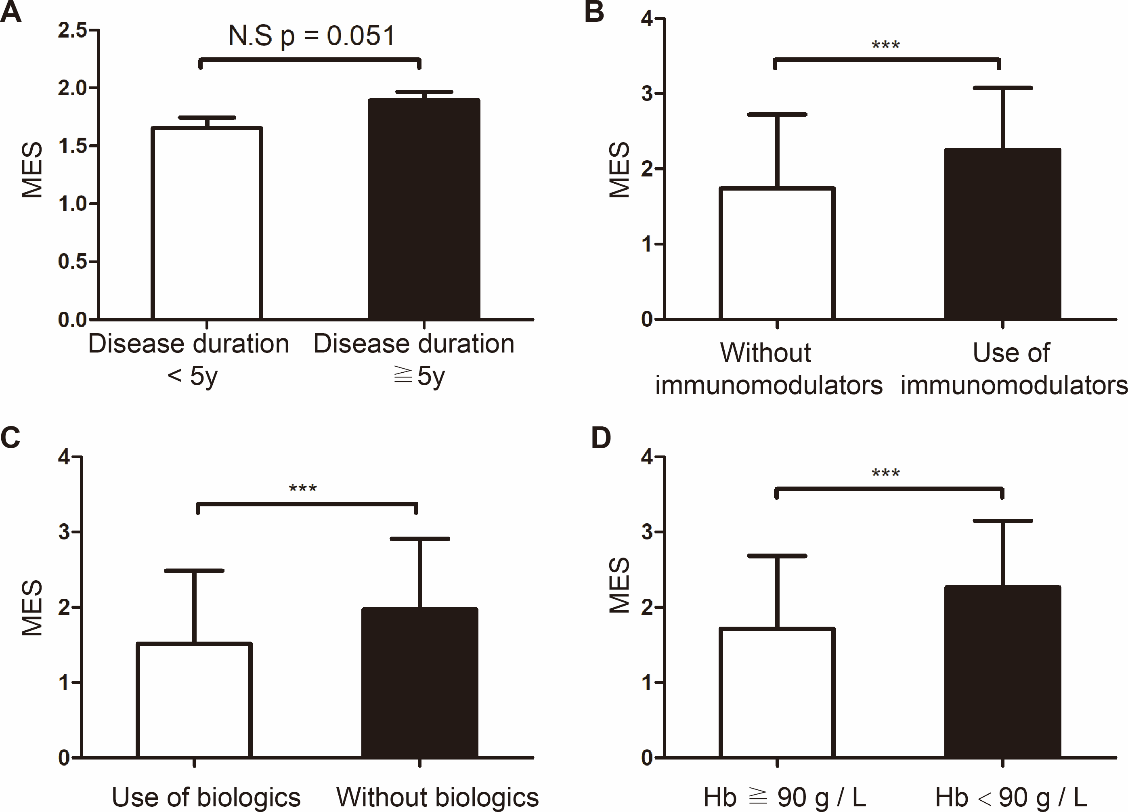


Supplementary Figure 1. Comparison of the MES in risk factors for High-MES by two-sample Student's t-test including (A) disease duration more than 5 years, (B) use of immunomodulators, (C) without biologics and (D) Hb < 90 g / L.


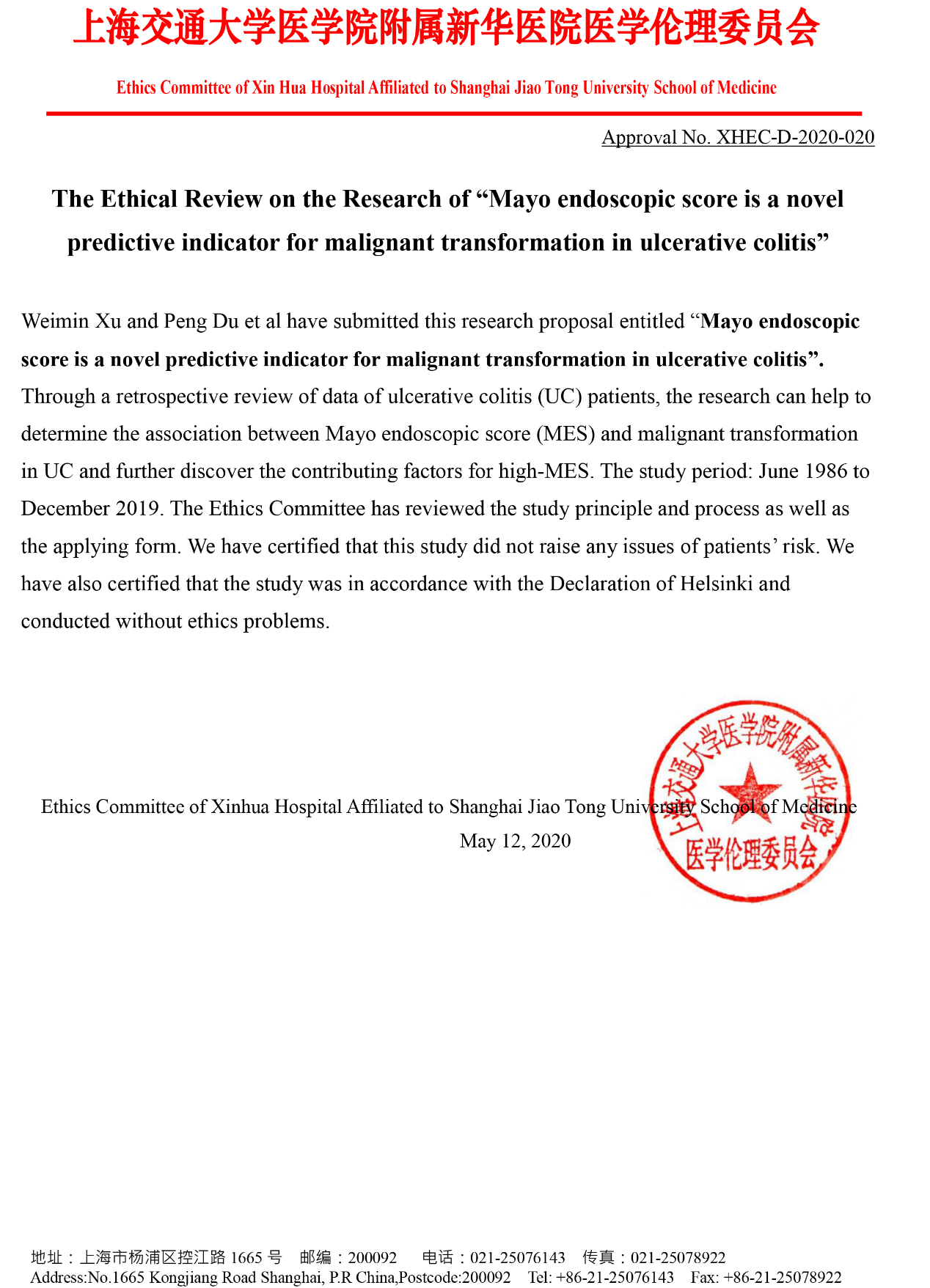


Ethical Approval for the study.


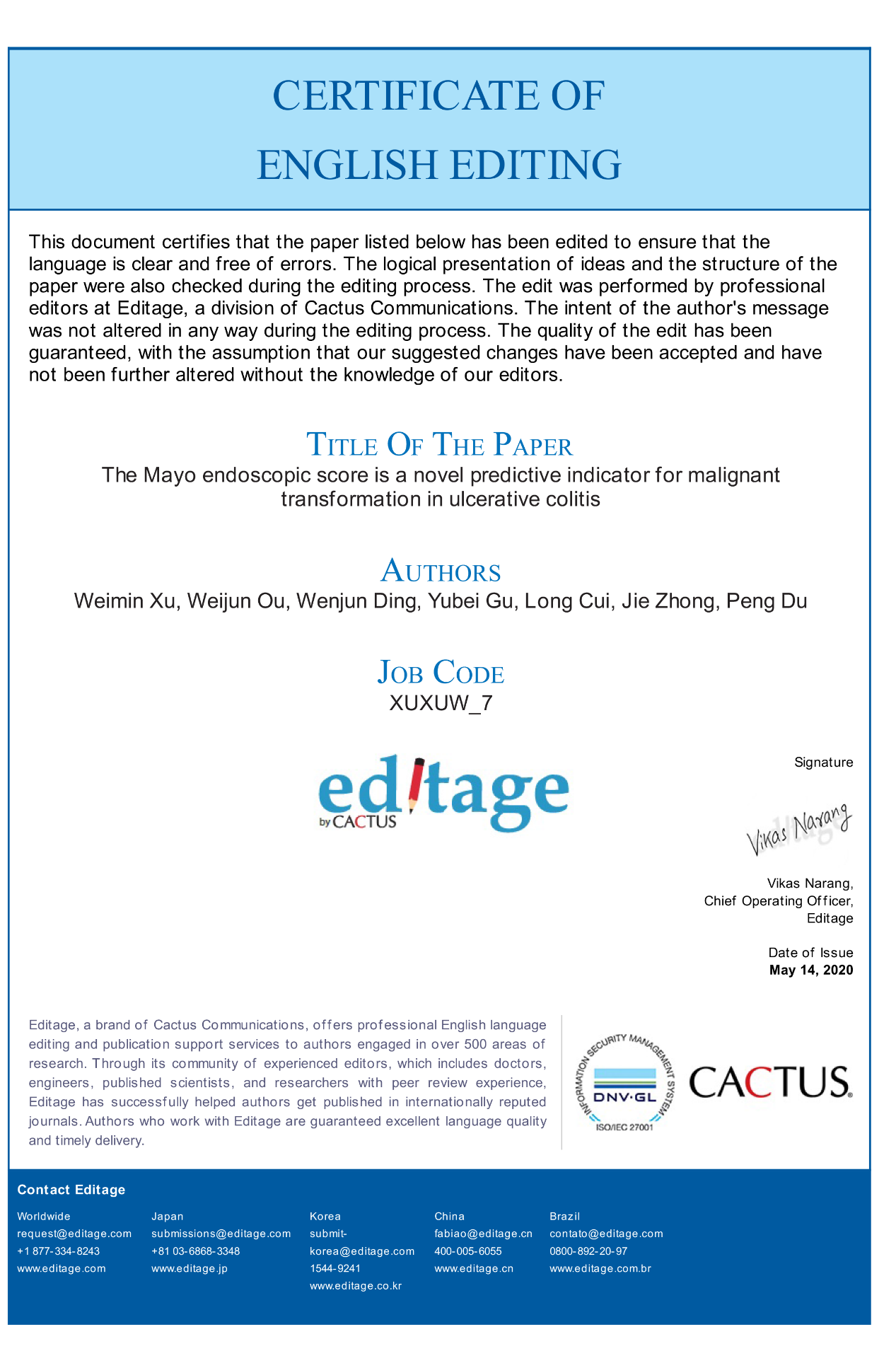


Certificate of English Editing for the study.
